# Disseminated histoplasmosis in a cat rescued in Fortaleza, Brazil, and successfully treated with itraconazole – First case report identified molecularly

**DOI:** 10.1016/j.mmcr.2020.09.005

**Published:** 2020-10-02

**Authors:** Liliane Lima da Silva Lomônaco, Stefanie Bressan Waller, Marlete Brum Cleff, Angelita dos Reis Gomes, Barbara Wilka Leal Silva, Rosane de Oliveira Cruz, Talles Monte de Almeida, Amanda Leal de Vasconcellos, Wesley Lyeverton Correia Ribeiro, Renata Osório de Faria, Mário Carlos Araújo Meireles, Adriana de Queiroz Pinheiro, Paula Priscila Correia Costa

**Affiliations:** a Veterinarian Autonomous, Fortaleza/CE, Brazil; b Faculty of Veterinary, Federal University of Pelotas, Rua Campus Universitario, 1, Pelotas/RS, 96010-900, Brazil; c Faculty of Veterinary, Ceará State University, Av. Dr. Silas Munguba, 1700, Campus Do Itaperi, Fortaleza/CE, 60714-903, Brazil; d Department of Physiology and Pharmacology, Federal University of Ceará, Rua Cel. Nunes de Melo, 1127, Rodolfo Teófilo, Fortaleza/CE, 6430-275, Brazil

## Abstract

An unneutered female cat of unknown age presented bloody lesions on the edematous face, and respiratory signs. Cytology and culture from the skin sample collected with fine-needle aspiration showed yeasts inside activated macrophages, and fungal growth characteristic of *Histoplasma* spp., which was molecularly confirmed that was *Histoplasma capsulatum* var. capsulatum. The cat was successfully treated with oral itraconazole (10 mg/kg/daily) for 120 days. This is the first case report of feline histoplasmosis confirmed molecularly in Brazil.

## 1 Introduction

Histoplasmosis is an important public health disease caused by the dimorphic fungus *Histoplasma* spp., which can be found in soil contaminated with bird or bat droppings [1]. This mycosis may affect both humans and domestic animals such as cats [1,2], and the main route of infection is through inhalation of fungal microconidia in the mycelial phase from contaminated areas [1]. Clinical signs of feline histoplasmosis included weight loss, anorexia, respiratory signs (dyspnea, tachypnea, cough, sneezing, wheezing) [[3], [4], [5], [6], [7], [8]], skin swelling or ulceration [4,5,9,10], lameness, vomiting [1], among other signs.

Feline histoplasmosis is considered endemic in United States [1,11], and there are a few reports in cats around the world, as in Italy [12], Germany [2], Thailand [13], Japan [14], Costa Rica [8], among other countries, but little is known about the cases in Brazil. According to Table 1, most of these reported cases occur in the state of Ceará [[3], [4], [5]], although no other cases have been found in the other Northeastern States. Additionally, histoplasmosis was reported in cats living in Southeastern Brazil, as in the states of Rio de Janeiro [6,7] and Minas Gerais [9], and in Southern Brazil, as in the Rio Grande do Sul [10,15], in which the fungal identifications were performed by phenotypic analysis by culture [[3], [4], [5], [6],^10^,^15^] or only cytology [7,9]. However, the feline cases of histoplasmosis in Brazil are underestimated, since the notification is not mandatory and, besides that, the diagnosis is often delayed or it is misdiagnosed due to the lack of specialized laboratories in mycological diagnosis in our reality.

**Table 1:** Feline cases of histoplasmosis in Brazil and their clinical and therapeutic approaches.

Brazilian city/State (*n*)	Sex	Age^a^	Clinical presentation	Previous history	Sample collection (Diagnosis)^b^	Antifungal therapy: dose (duration)^c^	Outcome	Reference
Fortaleza/CE (1)	F	Adult	Skin lesions, respiratory signs, anemia and lymphadenomegaly (disseminated)	Street rescued cat	FNAC (Cyt + Cult + Mol)	ITZ: 10 mg/kg, PO, q24h (for 4 m)	Resolution	Present study
Fortaleza/CE (1)	F	4y	Mild respiratory signs, increased nasal volume and enlarged submandibular lymph nodes	Contact with 24 cats with no lesions	FNAC (Cyt + Cult)	ITZ: 10 mg/kg, PO, q12h (for 2 m)	Resolution	[3]
Fortaleza/CE (1)	F	4y	Skin lesion, mild respiratory signs, increased nasal volume, enlarged submandibular lymph nodes	N.a.	N.a. (Cyt + Cult)	ITZ: 40 mg/ml, PO, q24h (for 21d); followed by ITZ (50 mg/pill, PO, q24h for 90d)	Resolution	[4]
Fortaleza/CE (1)	F	3y	Skin lesions, respiratory signs, increased nasal volume, and pneumonia (disseminated)	N.a.	FNAB (Cyt + Cult)	ITZ: 20 mg/kg, PO, q24h (for 3 m)	Resolution	[5]
Sobral/CE (1)	M	6y	Skin lesions, respiratory signs, increased nasal volume, lymphadenomegaly	Free outdoor access	Biopsy (Cult + Hist)	ITZ: 10 mg/kg, PO, q24h (for 21d)	Euthanasia	[5]
Baturité/CE (1)	M	3y	Skin lesions	Contact with fruit trees, plants, chickens, pigeons, goats, dogs and cats	Biopsy (Cult + Hist)	ITZ: 5 mg/kg, PO, q24h (for 60d)	No resolution and the cat died 20 days after the end of the therapy due possible FUS	[5]
Rio de Janeiro/RJ (1)	M	7y	Enlarged supraorbital region, conjunctivitis, respiratory signs and enlarged submandibular lymph nodes	Street rescued cat; corticoids for suspicion of allergic rhinitis	FNAC (Cyt + Cult)	ITZ: 10 mg/kg, PO, q24h (for 7d); followed by ITZ plus AMB (ITZ at same dose and AMB at 0.5 mg/kg, SC, 3 × /w for 2w); and, after, ITZ alone again (same previous dose for 6 m)	Resolution	[6]
Rio de Janeiro/RJ (1)	F	2y	Severe respiratory signs (pulmonary)	Free outdoor access in region of high density of pigeons and bats	Aspiration of pleural fluid (Cyt)	ITZ (100 mg/cat PO, q24h) plus AMB (0.5 mg/kg, SC, 16 applications at different intervals) at least 5 m	Resolution	[7]
Belo Horizonte/MG (1)	M	3y	Skin lesion, increased nasal volume, respiratory signs	Free outdoor access	Imprint (Cyt)	KTZ: 10 mg/kg, PO, q12h (for 15d) and continued at same dose, q24h (for 10d) plus KTZ topically	Resolution	[9]
São Francisco de Paula/RS (1)	F	2y	Skin lesions	No outdoor access; it was a rescued street cat	Biopsy (Cult + Hist)	ITZ: 5 mg/kg, PO, q12h	No resolution; the cat died after 1 week of treatment	[10]
Santa Maria/RS (1)	M	6y	Gastrointestinal signs, abdominal lymphadenomegaly, (disseminated)	Free outdoor access; habit of hunting pigeons	UGFNAC (Cyt + Cult)	ITZ: 5 mg/kg PO, q24h (for 138d), and changed to 10 mg/kg, PO, q24h (days 139–459)	Resolution	[15]

a y, year.

b FNAC, fine-needle aspiration cytology; FNAB, fine-needle aspiration biopsy; UGFNAC, ultrasound-guided fine-needle aspiration cytology (caudal mesenteric lymph node); Cyt, cytology; Cult, culture; Hist, histopathology; Mol, molecular.

c ITZ, itraconazole; AMB, amphotericin B; KTZ, ketoconazole; d, days; m, months; w, weeks; PO, per oral; SC, per subcutaneous; q12h, every 12 hours; q24h, every 24 hours; FUS, feline urological syndrome; N.a., Not available.

Treatment for histoplasmosis is based on the use of antifungal drugs, such as itraconazole, fluconazole, and amphotericin B, until lesions gradually regress [1,11]. The majority of cases in Brazil were resolved following antifungal therapy with itraconazole alone [[3], [4], [5], [6],^15^], or in combination with amphotericin B [7] or even with ketoconazole [9]. However, cases without resolution may evolve to either death or euthanasia [5,10], which further complicates the control of this disease.

This study aimed to report a case of disseminated histoplasmosis in a cat rescued in Fortaleza, Ceará (Northeast Brazil).

### 2 Case

**Fig. 1:**
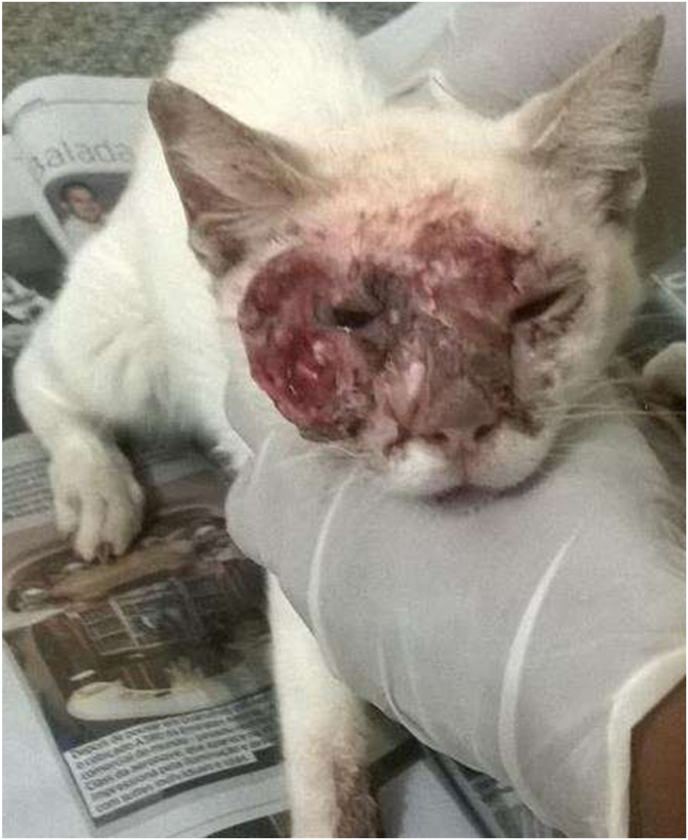
Cutaneous face lesions of a cat with disseminated histoplasmosis from Fortaleza, Ceará (Northeastern Brazil), before starting the antifungal treatment, showing edematous, ulcerative and hemorrhagic lesions.

An unspayed female cat of unknown adult age was rescued from a rural property where she was in contact with other cats and dogs. The new owner noticed ulcerative lesions on the animal's face two months prior, which worsened to the point that the cat was rescued and immediately taken to the veterinary clinic for evaluation on day zero. Physical examination revealed edematous and hemorrhagic lesions on the face, involving the right supra/infraorbital and nasal regions. A lesion was observed on the left region of the face, median to the eye (Fig. 1). The cat weighed 2.5 kg and showed apathy, mild diarrhea, mucopurulent nasal secretion, sneezes, moderate dehydration, enlarged submandibular lymph nodes, and subtle pulmonary crackles in lung auscultation. The ocular and oral mucous membranes were healthy pink and moist, and the body temperature was 38.2 °C (considered normal), without any indication of pain during abdominal palpation.

Blood samples were collected for complete blood count and serum biochemistry profile (alanine aminotransferase – ALT, and creatinine). After previously cleaning the area, skin lesions samples were collected by FNA and scrapings and swabs for cytology and microbiological cultures. Cytology using Romanowsky staining was performed with samples from ulcerative, erythematous, and hemorrhagic lesions from various areas on the face, mainly in infraorbital and supraorbital regions.

The results of the erythrogram showed mild non-regenerative anemia (4 × 10^6^/μl red blood cells; normal count: 5–10 × 10^6^ cells/μl); hemoglobin with 6.3 g/% (reference: 8–15 g/%) and hematocrit levels with 20% (reference: 24–45%), besides erythrocyte rouleaux and platelet aggregation. White blood cell count, ALT and creatinine values were within reference ranges. Although a new blood exam was recommended, the owner did not authorize further examination.

**Fig. 2:**
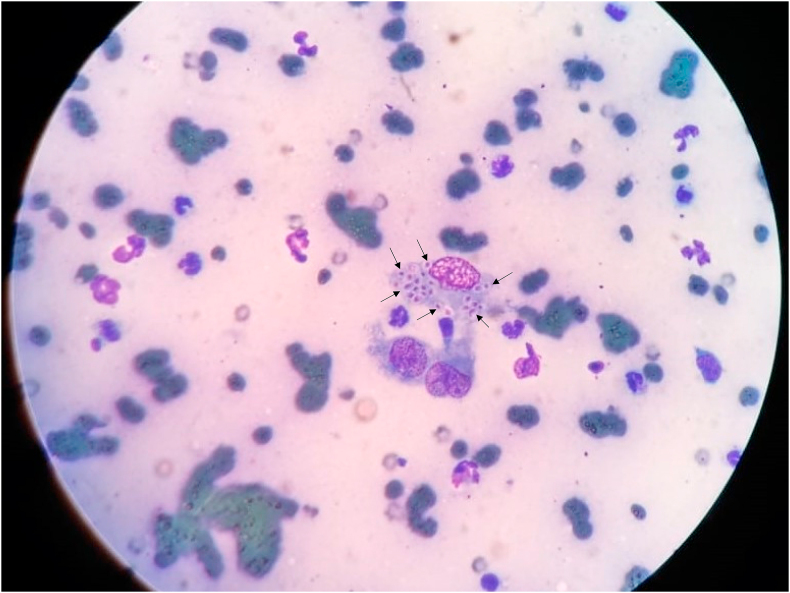
Direct smear by fine-needle aspiration (FNA) from skin lesions in the face of the cat, showing pyogranulomatous infiltrate and numerous yeast-like cells (arrows), suggestive of *Histoplasma capsulatum* var. *capsulatum* (Diff-Quick, 40 × objective).

Cytological findings showed hypercellularity of non-degenerate neutrophils. Moreover, activated macrophages showed intracytoplasmic structures featured as small-sized oval-to-round yeast with an extracellular surrounding halo and eccentric nucleus shaped as punctate or halfmoon (Fig. 2). Intracellular and extracellular coccoid bacteria were also noted, indicating a secondary infection. These results indicated a pyogranulomatous infiltrate with infection suggestive of *Histoplasma* spp.

Based on these results, treatment was initiated on the same day (day zero) with oral itraconazole (ITL 25mg®, 10 mg/kg, two times a day) along with the topical application of ketoconazole 2% (Navitae Ltda.®) in the lesions after cleaning with 0.9% NaCl solution. Considering the secondary infection, the cat was admitted for same day hospital treatment and received three intramuscular applications of amoxicillin trihydrate (Agemoxi L.A.®, 20 mg/kg/daily), every two days, along with a subcutaneous application of ketoprofen (Ketoflex® 1%, 2 mg/kg/daily), for four days.

Thoracic radiographs and abdominal ultrasound were performed on day 3 of treatment. The abnormal findings included lymphadenomegaly, that was observed in the lymph nodes of the gastroduodenal (0.78 cm), kidney (0.99 cm) and hepatic (0.95 cm) regions, in which the echotexture was slightly reduced and rounded, and an increase of the size liver, suggesting hepatic inflammatory process. Therefore, the frequency of administration of itraconazole was reduced at the same dose (10 mg/kg/daily), while the topical cream ketoconazole was stopped. A food supplement (Nutrisana Spirulina®, 2.5 mg/feed, daily) began on day 4 until the +100 days.

**Fig. 3:**
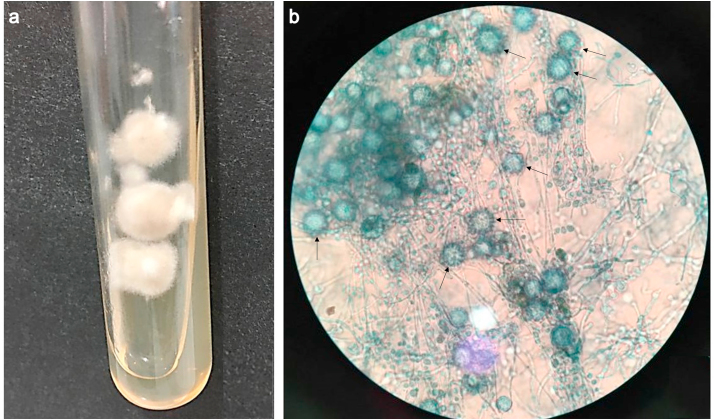
Fungal colony of *Histoplasma capsulatum* var. *capsulatum* isolated from the cutaneous lesions of the cat. Macroscopic characteristic in Mycosel agar on the 15th day of incubation at 37 °C (**a**), showing a cottony texture with a whitish color in the obverse and a brownish color on the reverse, and microscopic analysis (**b**) revealing hyaline septate hyphae containing rounded and spiculated macroconidia (arrows), characteristic of the pathogen (Lactophenol Cotton Blue, 40 × objective).

Considering that the gold standard for the diagnosis of histoplasmosis is the fungal culture associated with the reversion of the filamentous phase of the fungus to the yeast phase, samples of each clinical lesion collected by FNA was seeded on Saboraud, Saboraud plus chloramphenicol and Mycosel agar, and incubated at 25 °C in aerobiosis. On the 15th day of incubation, all culture media showed fungal growth of a whitish cottony texture on the obverse (Fig. 3a) and brownish color on the reverse. Microscopically, hyaline septate hyphae and rounded and spiculated macroconidia were observed at a magnification of 400 × , from 8 to 15 μm (Fig. 3b). For the filamentous phase reversion to yeast, colonies were seeded on BHI agar (Brain Heart Infusion) plus 5% sheep blood and incubated at 35 °C. The phase reversal showed a bright and creamy colony, microscopically characterized by small oval yeasts (1–5 μm in diameter), confirming the fungal dimorphism. Due to biological risk, handling of the pathogen was carefully performed in a laminar flow hood.

Fragments of the fungal colony obtained from the culture were submitted to DNA extraction following molecular diagnostic by the polymerase chain reaction (PCR) in order to confirm the pathogenic agent. The *RYP1* gene (263 bp) was amplified using the following primers OAS1057 (5′-ACC CTT GCA GCT TAC AAC CT-3′) and OAS1058 (5′-TCC GTC CAT CGC TTA ATA CC-3 ′), besides specific amplicons (300 bp) to *RYP1* genes, according to Nguyen and Sil [16]. The amplified products were analyzed by electrophoresis on 1% agar gel using Blue Green (Loading Dye) as a fluorescent dye and visualized in a UV-transilluminator. Negative and positive controls were used from a human histoplasmosis case. The fungal sample from the feline case was identified as *Histoplasma capsulatum* var. *capsulatum*. The sequence of this isolate was previously deposited online on Genbank (number MK893850.1) and it was identified by data bank analysis with NCBI BLAST, in which presented between 97.44% and 100% identity with 102 human clinical isolates and one feline isolate.

**Fig. 4:**
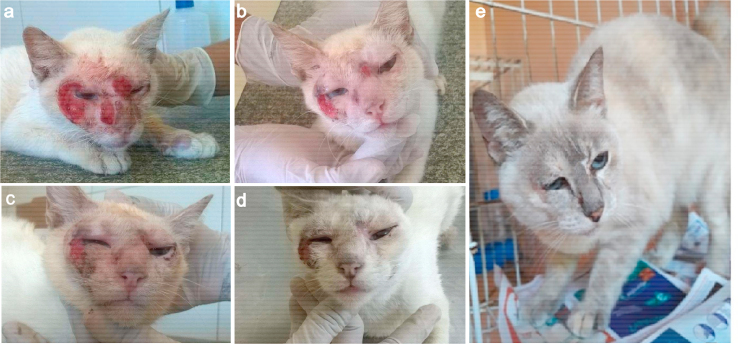
Evolution of the regression of the cutaneous lesions on the face of a cat with disseminated histoplasmosis during antifungal treatment with itraconazole at the following days of treatment: day 4 (**a**), day 9 (**b**), day 18 (**c**), day 23 (**d**) and day 120 (**e**).

The antifungal treatment with itraconazole was performed for 120 days, and the patient showed improvement of clinical signs from the first 30 days of therapy. No issued of vomiting or diarrhea were noted during the treatment. The evolution of the clinical treatment showed the complete remission of the cutaneous lesions until the final period of treatment (Fig. 4). Although no new imaging exams were performed due to the financial condition of the owner, the cat became pregnant one year after this episode and gave birth to healthy kittens. Afterward, the cat was spayed and has been living a healthy life without any recurrences.

## 3 Discussion

The feline patient presented anemia, respiratory sounds, nasal secretion, dyspnea, intra-abdominal lymphadenopathy, and cutaneous lesions. These results corroborate with previous authors [1,[3], [4], [5], [6], [7],^9^,^10^], who confirm that animals with this condition may present lymphadenomegaly, weight loss, inappetence, and respiratory signs, such as light chronic coughing, dyspnea, and tachypnea.

Results of the hematological profile showed mild anemia and the serum biochemical exam was within normal standards. According to some authors, non-regenerative normocytic-normochromic anemia [5,7,10,12,15] may occur in these cases with a normal white cell count [1,5,9,10,13]. Other studies also described that elevated ALT activity can be observed [1,12,14], which did not occur in this case. Although a new blood exam was recommended, the owner did not authorize it. Thoracic radiographs and abdominal ultrasounds are recommended for evaluating the increase or decrease of the organs and, in our case, the cat had mild evidence of a hepatopathy and mesenteric lymph node enlargement. Additionally, histoplasmosis can be diagnosed using antigen detection on serum or urine [1], although this antigen detection assay was not performed in the reported feline.

The cases of histoplasmosis are regional in their distribution [1,5]. It is known that *H. capsulatum* is endemic throughout large areas of the temperate and subtropical regions of the world and tends to prefer areas with warm (mean ambient temperature of 22 °C to 29 °C) and humid conditions, which are generally between latitudes 45° north and 30° south [1]. The feline patient came from Fortaleza (Ceará, Northeastern Brazil), a geographical region where several feline cases have been reported [[3], [4], [5]]. This city is characterized by a rainy tropical climate, ranging from humid to sub-humid [17], providing climatic conditions in which should be taken into consideration during the patient's clinical investigation. The present case of feline histoplasmosis was confirmed after cytology, fungal culture, and molecular identification.

Similar cutaneous lesions in cats may be caused by fungal pathogens belonging to the clinical clades of *Sporothrix* spp. and *Cryptococcus* spp. complex [2], as well as by feline eosinophilic granuloma or squamous cell carcinoma [18]. Both *Sporothrix* spp. species and *Histoplasma* spp. may appear as small, round, or oval cells under direct microscopic examination, but only *Sporothrix* spp. show cigar-shaped yeast cells. Considering that the felines cases of sporotrichosis are often and this fungal genus is also dimorphic, like *Histoplasma* spp., a definitive diagnosis requires a fungal culture [18,19].

In the *Cryptococcus* spp. complex, yeast cells are round, commonly deeply basophilic, with a round, oval or elliptical nucleus and a characteristically wide mucopolysaccharide capsule that does not stain with the common rapid Romanowsky-type dyes. These thick non-staining capsules, occasionally with narrow-based budding, differ from the *Histoplasma* spp. yeast cells that have a thin poorly stained cell wall [19]. Additionally, the cryptococcosis cases in animals can be easily diagnosed by immunochromatography with latex antigen agglutination testing in serum specimens [20].

In our patient, the cytological findings revealed a pyogranulomatous inflammatory process with unaltered and degenerated neutrophils and activated macrophages. Inside these cells, oval-to-round structures suggestive of *H. capsulatum* were identified.

According to certain authors [1,18], the quickest method to collect samples in these cases is by scraping and FNA. They also affirm that Diff-Quick is the ideal staining option to identify yeast structures of *Histoplasma* spp. (Fig. 2). These results corroborate with studies [1], which affirm that this pathogen may be observed in cytology [[3], [4], [5], [6], [7],^9^,^12^,^14^,^15^], biopsy [5,10,13] or culture [[3], [4], [5], [6],^8^,^10^,^15^]. The importance of cytology in obtaining a rapid diagnosis is emphasized, thus providing greater speed to introduce the appropriate therapy for the case. Additionally, it is a quick, practical, less invasive, and cheaper examination compared to histopathology [18].

Direct examination, fungal culture, and phase fungal reversal were fundamental for the confirmation and diagnosis of histoplasmosis in our case. The diagnosis made by culture mediums (Sabouraud agar, BHI agar, Potato Dextrose agar, Lactrimel agar, and Malt agar) [19] is considered as the gold standard for histoplasmosis diagnosis [[3], [4], [5], [6],^8^,^10^,^15^], but the manipulation of the agent must be carefully performed in a safety cabinet due to the pathogenic potential [1].

However, one disadvantage lies in the difficulty of microscopically recognizing histoplasmosis by non-experienced mycologists, since other fungi may also present tuberculated macroconidia which appear as similar to *Histoplasma* spp., and may be mistaken for *Chrysosporium* spp. and *Sepedonium* spp [19]. At specific temperatures, only *H. capsulatum* assumes distinct forms. Under ambient temperature, a morphological macro colony of cottony appearance and micro morphologically, the mycelial form is constituted of hyaline hyphae, septate and branched, with smooth microconidia, which is the infecting form and tuberculated macroconidia. At 37 °C, it appears as a macroscopically bright and creamy colony and is microscopically characterized by small oval yeasts (about 1–5 μm in diameter) [1,19].

Regarding the antifungal options for histoplasmosis treatment in cats, itraconazole alone [[3], [4], [5], [6],^15^] or associated with amphotericin B [7,13] are recommended [1,11]. Although cases with no resolution using itraconazole have been reported [5,10,12], this antifungal agent is most common for therapy with good response [[3], [4], [5], [6],^11^,^13^,^15^], where the median duration in 101 cats with histoplasmosis was 137 days [11]. This finding corroborates with the treatment duration of our case. The current rescued cat showed complete remission of the cutaneous lesions after 120 days of antifungal treatment.

We reported a feline case of disseminated histoplasmosis in Fortaleza (Ceará, Northeastern Brazil), an endemic region where cases of this disease in cats is increasing. This study highlighted the importance of cytology as a fast, simple, and low-cost diagnostic auxiliary tool in veterinary routine, allowing for the rapid introduction of antifungal therapy, followed by the confirmation of the infection by *H. capsulatum* var. *capsulatum* through fungal culture and molecular identification. The molecular identification by PCR for amplification of the *RYP1* gene allowed confirmation of the pathogen and, to our knowledge, this is the first case report of feline histoplasmosis confirmed molecularly in Brazil. The correct identification of the etiological agent is essential for therapeutic success, where the cat showed complete remission of the cutaneous lesions after 120 days of therapy with oral itraconazole, without any adverse effects. Additionally, the evolutionary follow-up of antifungal treatment has been demonstrated in this study.

## Ethical form

The authors declare that the consent was obtained from the patient's owner.

## Declaration of competing interest

There are none.
